# The genome sequence of the greater argentine, *Argentina silus *(Ascanius, 1775)

**DOI:** 10.12688/wellcomeopenres.23691.1

**Published:** 2025-03-17

**Authors:** Helga Bára Mohr Vang, Ian Salter, Sunnvør í Kongsstovu, Svein-Ole Mikalsen, Wellcome Sanger Institute Tree of Life Management, Samples and Laboratory team, Wellcome Sanger Institute Scientific Operations: Sequencing Operations, Wellcome Sanger Institute Tree of Life Core Informatics team, Tree of Life Core Informatics collective

**Affiliations:** 1 Faroe Marine Research Institute, Tórshavn, Streymoy, Faroe Islands; 2 Faculty of Science and Technology, University of the Faroe Islands, Tórshavn, Streymoy, Faroe Islands

**Keywords:** Argentina silus, greater argentine, genome sequence, chromosomal, Argentiniformes

## Abstract

We present a genome assembly from a male specimen of *Argentina silus* (the greater argentine; Chordata; Actinopteri; Argentiniformes; Argentinidae). The genome sequence has a total length of 670.70 megabases. Most of the assembly is scaffolded into 24 chromosomal pseudomolecules, including the X and Y sex chromosomes. The mitochondrial genome has also been assembled and is 16.64 kilobases in length. Gene annotation of this assembly on Ensembl identified 19,422 protein-coding genes.

## Species taxonomy

Eukaryota; Opisthokonta; Metazoa; Eumetazoa; Bilateria; Deuterostomia; Chordata; Craniata; Vertebrata; Gnathostomata; Teleostomi; Euteleostomi; Actinopterygii; Actinopteri; Neopterygii; Teleostei; Osteoglossocephalai; Clupeocephala; Euteleosteomorpha; Protacanthopterygii; Argentiniformes; Argentinidae; *Argentina*; *Argentina silus* (Ascanius, 1775) (NCBI:txid446415).

## Background

**Figure 1.**
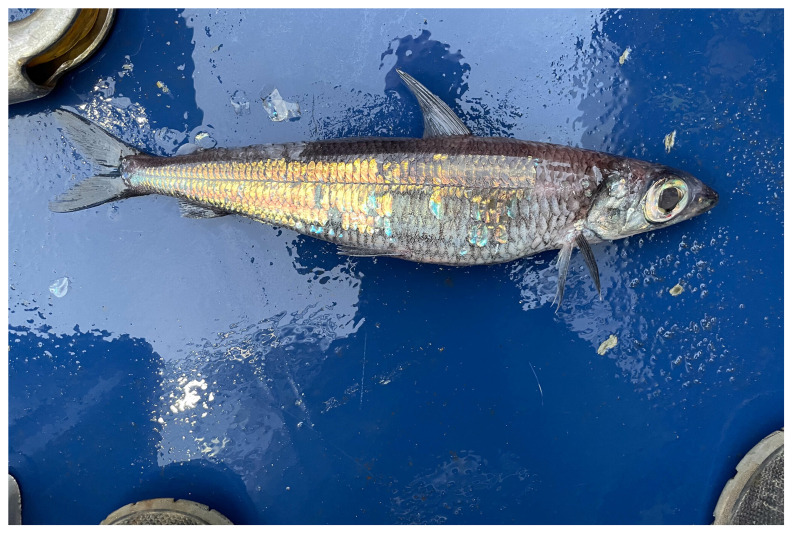
*Argentina silus* (Photograph by Claude Nozères CC BY-NC).

The greater silver smelt ( *Argentina silus*) ( Figure 1) is an important part of the ecosystem and biodiversity in the North Atlantic Ocean. This benthopelagic fish inhabits depths ranging from 150–1400 m. It is a long-lived, slow-growing species with a broad geographical distribution across the North-Atlantic Ocean ( Coad & Reist, 2004; Mecklenburg *et al.*, 2011). Species with such characteristics are particularly vulnerable to overexploitation, as their slow growth and delayed maturity limit their ability to recover population size following periods of high mortality. Therefore, effective monitoring and management of fisheries targeting this species are essential to ensure sustainability.

The greater silver smelt has become a valuable resource for several North Atlantic nations during the last 30 to 40 years, with average total catches estimated at around 20,000 tonnes ( ICES, 2022). Despite its economic importance, the population structure of the greater silver smelt remains poorly understood. In 2015, the International Council for the Exploration of the Sea advised that the greater silver smelt should be divided into four assessment units ( ICES, 2014). However, it is unclear whether these assessment units correspond to distinct biological populations. Studies using parasites ( Scott, 1969) and mitochondrial DNA sequencing ( Árnason, 2017) did not detect population structure. In contrast, a recent study by Quintela *et al.* (2024), using ddRAD sequencing and a SNP panel suggested that at least one management unit may consist of more than one biological population. With the present chromosomal-level genome assembly and subsequent whole-genome resequencing, the Gen@FarE project aims to achieve a high-resolution analysis of the population structure of the greater silver smelt in the Northeast Atlantic Ocean. This information will provide valuable insights to support the sustainable management of this species.

## Genome sequence report

The genome of a male *Argentina silus* was sequenced using Pacific Biosciences single-molecule HiFi long reads, generating a total of 43.48 Gb (gigabases) from 4.38 million reads, providing an estimated 84-fold coverage. Primary assembly contigs were scaffolded with chromosome conformation Hi-C data, which produced 134.66 Gb from 891.80 million reads, yielding an approximate coverage of 201-fold. Specimen and sequencing details are provided in Table 1.

**Table 1.** Specimen and sequencing data for *Argentina silus*.

Project information			
**Study title**	Argentina silus (greater argentine)		
**Umbrella BioProject**	PRJEB59966		
**Species**	*Argentina silus*		
**BioSample**	SAMEA110137623		
**NCBI taxonomy ID**	446415		
Specimen information			
**Technology**	**ToLID**	**BioSample ** **accession**	**Organism ** **part**
**PacBio long read sequencing**	fArgSil1	SAMEA110137641	Gill
**Hi-C sequencing**	fArgSil1	SAMEA110137633	Gill
**RNA sequencing**	fArgSil1	SAMEA110137642	Gill
Sequencing information			
**Platform**	**Run accession**	**Read count**	**Base count (Gb)**
**Hi-C Illumina NovaSeq 6000**	ERR10908640	8.92e+08	134.66
**PacBio Sequel IIe**	ERR10906104	5.72e+05	4.88
**PacBio Sequel IIe**	ERR10906103	2.13e+06	21.89
**PacBio Sequel IIe**	ERR10906102	1.67e+06	16.71
**RNA Illumina NovaSeq 6000**	ERR10908641	7.04e+07	10.63

Assembly errors, including 63 missing joins or mis-joins and five haplotypic duplications, were corrected by manual curation. This reduced the scaffold number by 2.94% and increased the scaffold N50 by 4.34%. The final assembly has a total length of 670.70 Mb in 1,023 sequence scaffolds, with 1,752 gaps, and a scaffold N50 of 24.5 Mb ( Table 2).

**Table 2.** Genome assembly data for *Argentina silus*, fArgSil1.1.

Genome assembly		
Assembly name	fArgSil1.1	
Assembly accession	GCA_951799395.1	
*Accession of alternate haplotype*	*GCA_951799415.1*	
Span (Mb)	670.70	
Number of contigs	2,776	
Contig N50 length (Mb)	0.9	
Number of scaffolds	1,023	
Scaffold N50 length (Mb)	24.5	
Longest scaffold (Mb)	54.1	
Assembly metrics *		*Benchmark*
Consensus quality (QV)	52.5	*≥ 50*
*k*-mer completeness	Combined: 99.30% (primary 90.22%; alternate: 78.73%).	*≥ 95%*
BUSCO **	C:96.1%[S:93.7%,D:2.4%], F:1.6%,M:2.3%,n:3,640	*C ≥ 95%*
Percentage of assembly mapped to chromosomes	92.32%	*≥ 95%*
Sex chromosomes	XY	*localised homologous pairs*
Organelles	Mitochondrial genome: 16.64 kb	*complete single alleles*
Genome annotation of assembly GCA_951799395.1 at Ensembl		
Number of protein-coding genes	19,422	
Number of non-coding genes	11,082	
Number of gene transcripts	48,785	

* Assembly metric benchmarks are adapted from column VGP-2020 of “Table 1: Proposed standards and metrics for defining genome assembly quality” from Rhie *et al.* (2021). ** BUSCO scores based on the actinopterygii_odb10 BUSCO set using version 5.3.2. C = complete [S = single copy, D = duplicated], F = fragmented, M = missing, n = number of orthologues in comparison. A full set of BUSCO scores is available at https://blobtoolkit.genomehubs.org/view/fArgSil1_1/dataset/fArgSil1_1/busco.

**Figure 2.**
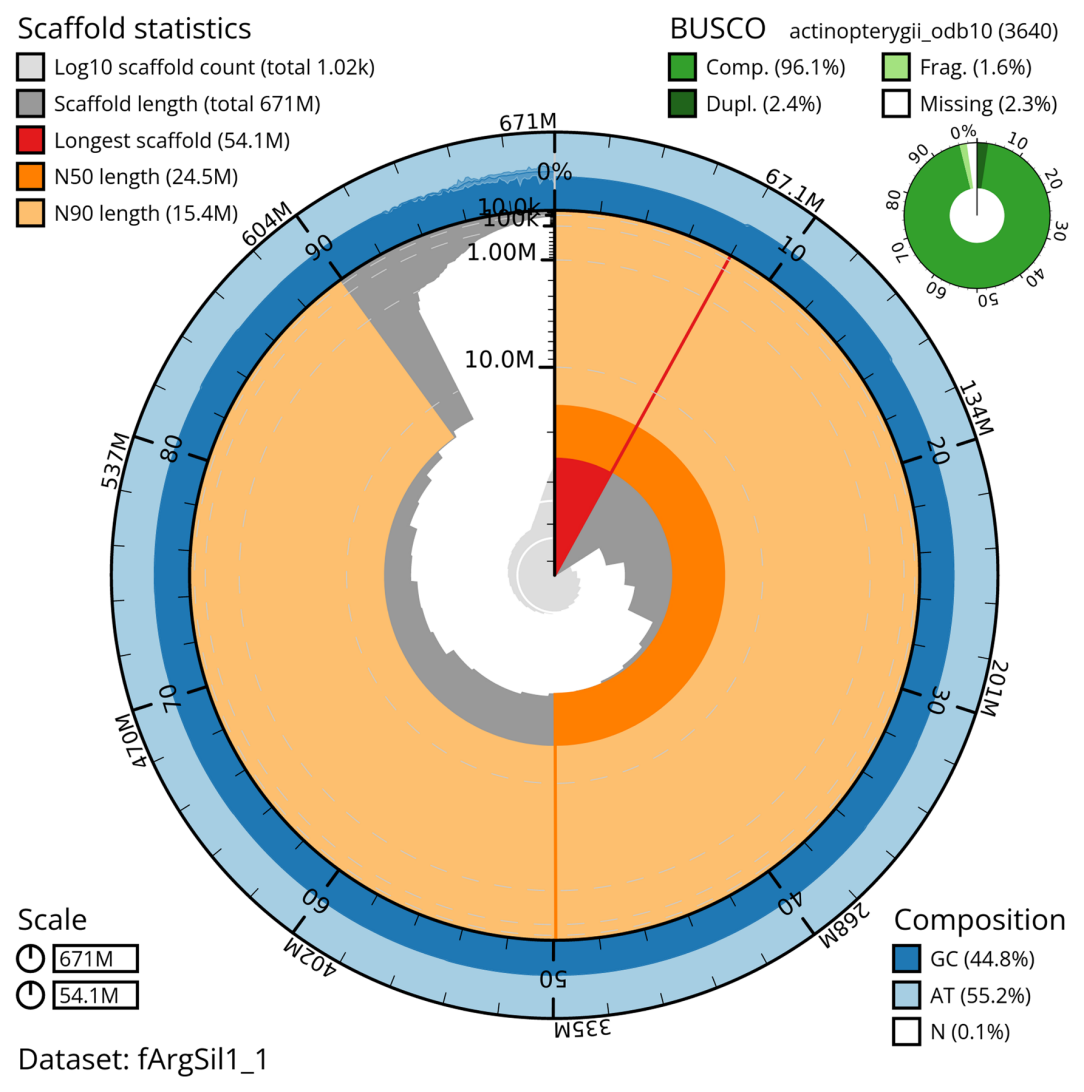
Genome assembly of *Argentina silus*, fArgSil1.1: metrics. The BlobToolKit snail plot shows N50 metrics and BUSCO gene completeness. The main plot is divided into 1,000 size-ordered bins around the circumference with each bin representing 0.1% of the 670,765,936 bp assembly. The distribution of scaffold lengths is shown in dark grey with the plot radius scaled to the longest scaffold present in the assembly (54,096,584 bp, shown in red). Orange and pale-orange arcs show the N50 and N90 scaffold lengths (24,540,466 and 15,361,704 bp), respectively. The pale grey spiral shows the cumulative scaffold count on a log scale with white scale lines showing successive orders of magnitude. The blue and pale-blue area around the outside of the plot shows the distribution of GC, AT and N percentages in the same bins as the inner plot. A summary of complete, fragmented, duplicated and missing BUSCO genes in the actinopterygii_odb10 set is shown in the top right. An interactive version of this figure is available at https://blobtoolkit.genomehubs.org/view/fArgSil1_1/dataset/fArgSil1_1/snail.

**Figure 3.**
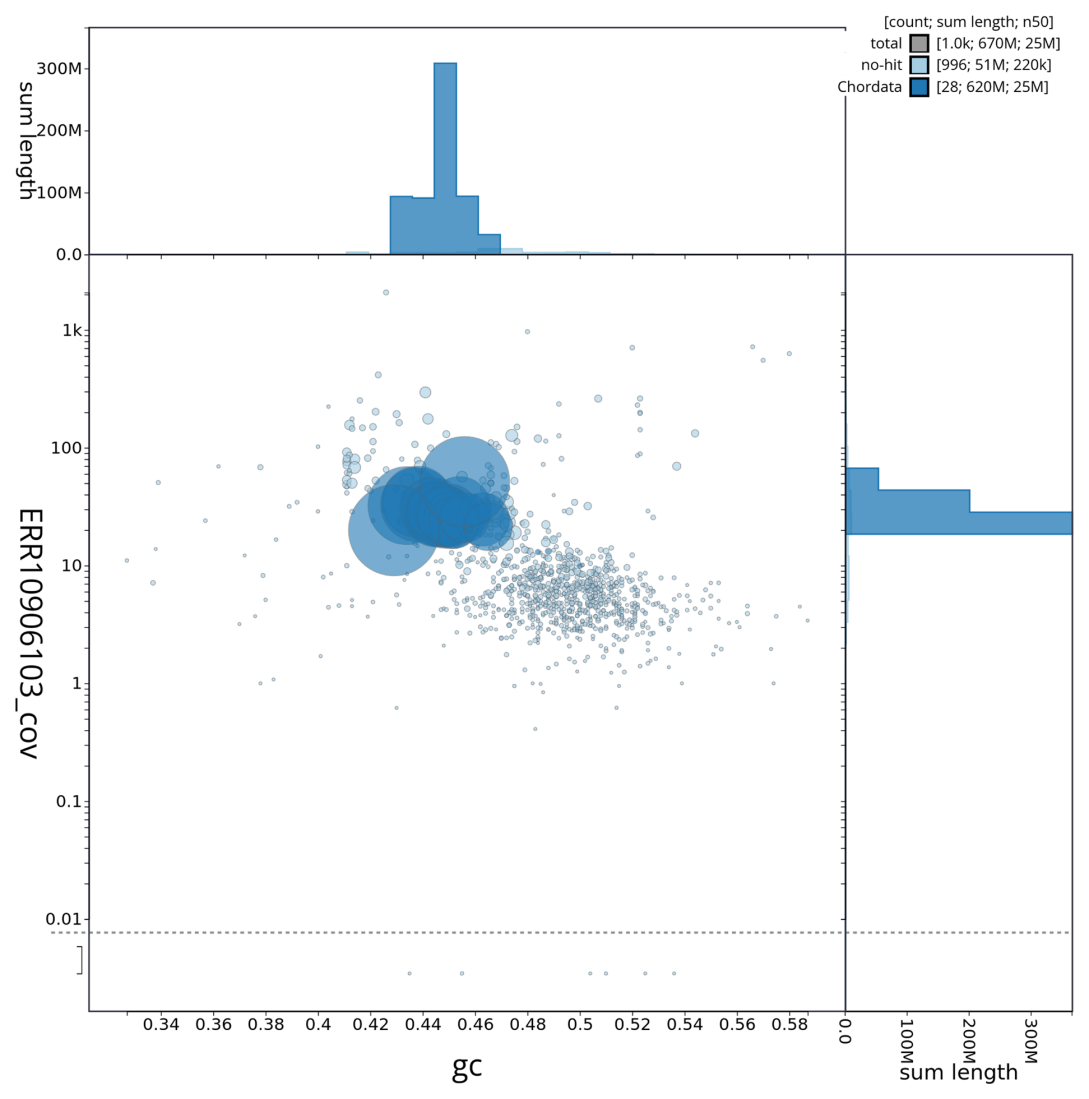
Genome assembly of *Argentina silus*, fArgSil1.1: BlobToolKit GC-coverage plot showing sequence coverage (vertical axis) and GC content (horizontal axis). The circles represent scaffolds, with the size proportional to scaffold length and the colour representing phylum membership. The histograms along the axes display the total length of sequences distributed across different levels of coverage and GC content. An interactive version of this figure is available at https://blobtoolkit.genomehubs.org/view/fArgSil1_1/dataset/fArgSil1_1/blob.

**Figure 4.**
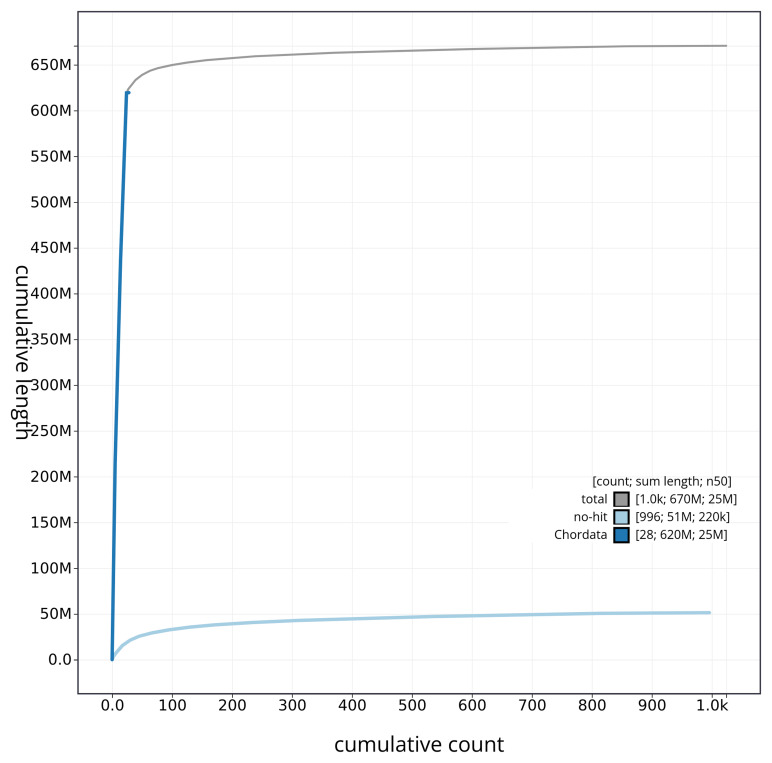
Genome assembly of *Argentina silus* fArgSil1.1: BlobToolKit cumulative sequence plot. The grey line shows cumulative length for all sequences. Coloured lines show cumulative lengths of sequences assigned to each phylum using the buscogenes taxrule. An interactive version of this figure is available at https://blobtoolkit.genomehubs.org/view/fArgSil1_1/dataset/fArgSil1_1/cumulative.

The snail plot in Figure 2 provides a summary of the assembly statistics, indicating the distribution of scaffold lengths and other assembly metrics. Figure 3 shows the distribution of scaffolds by GC proportion and coverage. Figure 4 presents a cumulative assembly plot, with separate curves representing different scaffold subsets assigned to various phyla, illustrating the completeness of the assembly.

The snail plot in Figure 2 provides a summary of the assembly statistics, while the distribution of assembly scaffolds on GC proportion and coverage is shown in Figure 3. The cumulative assembly plot in Figure 4 shows curves for subsets of scaffolds assigned to different phyla.

**Figure 5.**
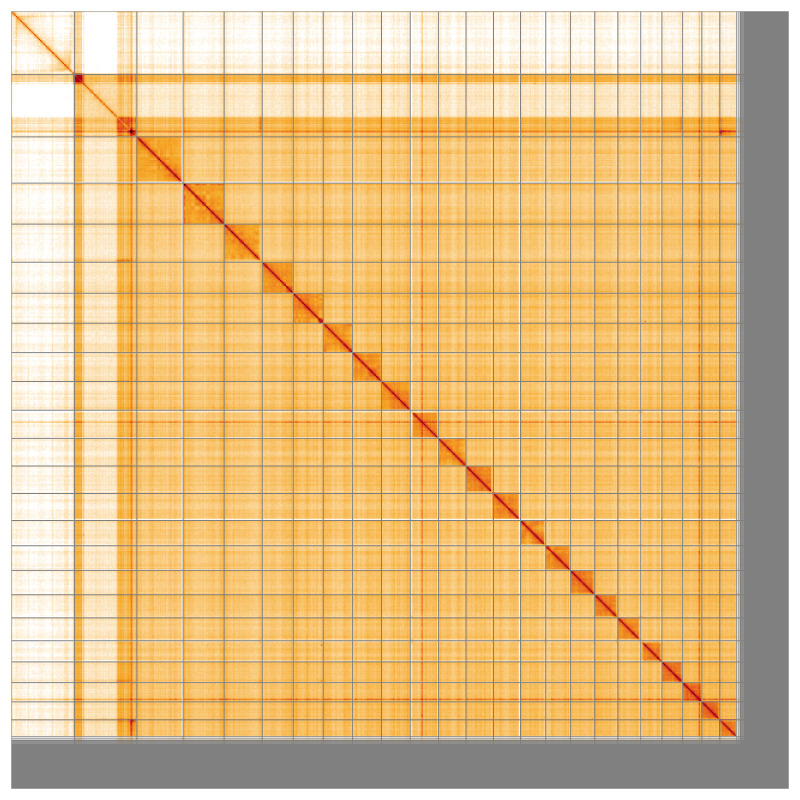
Genome assembly of *Argentina silus* fArgSil1.1: Hi-C contact map of the fArgSil1.1 assembly, visualised using HiGlass. Chromosomes are shown in order of size from left to right and top to bottom. An interactive version of this figure may be viewed at https://genome-note-higlass.tol.sanger.ac.uk/l/?d=eyZ0NUKYR1agttl3NllTuQ.

Most of the assembly sequence (92.32%) was assigned to 24 chromosomal-level scaffolds, representing 22 autosomes and the X and Y sex chromosomes. Chromosome-scale scaffolds confirmed by the Hi-C data are named in order of size ( Figure 5; Table 3). During manual curation, sex chromosomes were annotated based on coverage statistics as well as synteny to the assembly of *Gasterosteus aculeatus aculeatus* (GCF_016920845.1).

While not fully phased, the assembly deposited is of one haplotype. Contigs corresponding to an alternate haplotype have also been deposited. The mitochondrial genome was also assembled and can be found as a contig within the multifasta file of the genome submission.

The estimated Quality Value (QV) of the final assembly is 52.5. The *k*-mer completeness for the combined primary and alternate haplotypes is 99.30% (primary 90.22%; alternate: 78.73%). The assembly has a BUSCO v5.3.2 completeness of 96.1% (single = 93.7%, duplicated = 2.4%), using the actinopterygii_odb10 reference set ( *n* = 3,640).

**Table 3.** Chromosomal pseudomolecules in the genome assembly of *Argentina silus*, fArgSil1.

INSDC accession	Name	Length (Mb)	GC%
OX637368.1	1	39.47	43.5
OX637369.1	2	35.03	44.0
OX637370.1	3	32.54	44.0
OX637371.1	4	26.48	44.5
OX637372.1	5	25.59	45.5
OX637373.1	6	25.08	44.5
OX637374.1	7	24.67	45.0
OX637375.1	8	24.54	45.0
OX637376.1	9	24.0	45.0
OX637377.1	10	23.53	44.5
OX637378.1	11	23.52	44.5
OX637379.1	12	23.08	44.5
OX637380.1	13	21.48	45.0
OX637381.1	14	21.04	44.5
OX637382.1	15	20.86	45.0
OX637383.1	16	19.73	45.0
OX637384.1	17	19.47	45.0
OX637385.1	18	18.04	45.0
OX637386.1	19	17.69	46.5
OX637387.1	20	16.51	45.0
OX637388.1	21	15.36	45.5
OX637389.1	22	14.38	46.5
OX637366.1	X	54.1	43.0
OX637367.1	Y	53.08	45.5
OX637390.1	MT	0.02	48.5

## Genome annotation report

The *Argentina silus* genome assembly (GCA_951799395.1) was annotated at the European Bioinformatics Institute (EBI) on Ensembl Rapid Release. The resulting annotation includes 48,785 transcribed mRNAs from 19,422 protein-coding and 11,082 non-coding genes ( Table 2; https://rapid.ensembl.org/Argentina_silus_GCA_951799395.1/Info/Index). The average transcript length is 11,834.22. There are 1.60 coding transcripts per gene and 10.33 exons per transcript.

## Methods

### Sample acquisition

An adult male specimen of *Argentina silus* (specimen ID ERGA SM FO 02, ToLID fArgSil1) was collected aboard R/V Jákup Sverri at Faroe Bank (latitude 61.13, longitude –9.35) on 2021-09-12. The specimen was collected and identified by Helga Bára Mohr Vang (Faroe Marine Research Institute). The specimen was collected and identified by Helga Bára Mohr Vang (Faroe Marine Research Institute). The following biological parameters were recorded for the specimen: length, weight, life stage, and sex. Tissue samples were collected from the gills, pectoral fin, and caudal fin, and immediately frozen in liquid nitrogen by Ian Salter (Faroe Marine Research Institute). The remaining portion of the specimen was preserved in 96% ethanol if further analyses were needed.

### Nucleic acid extraction

The workflow for high molecular weight (HMW) DNA extraction at the Wellcome Sanger Institute (WSI) Tree of Life Core Laboratory includes a sequence of core procedures: sample preparation and homogenisation, DNA extraction, fragmentation and purification. Detailed protocols are available on protocols.io ( Denton *et al.*, 2023b). The fArgSil1 sample was prepared for DNA extraction by weighing and dissecting it on dry ice ( Jay *et al.*, 2023), and tissue from the gills was homogenised using a PowerMasher II tissue disruptor ( Denton *et al.*, 2023a).

HMW DNA was extracted using the Automated MagAttract v1 protocol ( Sheerin *et al.*, 2023). DNA was sheared into an average fragment size of 12–20 kb in a Megaruptor 3 system ( Todorovic *et al.*, 2023). Sheared DNA was purified by solid-phase reversible immobilisation, using AMPure PB beads to sample to eliminate shorter fragments and concentrate the DNA ( Strickland *et al.*, 2023). The concentration of the sheared and purified DNA was assessed using a Nanodrop spectrophotometer and Qubit Fluorometer using the Qubit dsDNA High Sensitivity Assay kit. Fragment size distribution was evaluated by running the sample on the FemtoPulse system.

RNA was extracted from gill tissue of fArgSil1 in the Tree of Life Laboratory at the WSI using the RNA Extraction: Automated MagMax™ *mir*Vana protocol ( do Amaral *et al.*, 2023). The RNA concentration was assessed using a Nanodrop spectrophotometer and a Qubit Fluorometer using the Qubit RNA Broad-Range Assay kit. Analysis of the integrity of the RNA was done using the Agilent RNA 6000 Pico Kit and Eukaryotic Total RNA assay.

### Hi-C preparation

Gill tissue of the fArgSil1 sample was processed at the WSI Scientific Operations core, using the Arima-HiC v2 kit. Tissue (stored at –80 °C) was fixed, and the DNA crosslinked using a TC buffer with 22% formaldehyde. After crosslinking, the tissue was homogenised using the Diagnocine Power Masher-II and BioMasher-II tubes and pestles. Following the kit manufacturer's instructions, crosslinked DNA was digested using a restriction enzyme master mix. The 5’-overhangs were then filled in and labelled with biotinylated nucleotides and proximally ligated. An overnight incubation was carried out for enzymes to digest remaining proteins and for crosslinks to reverse. A clean up was performed with SPRIselect beads prior to library preparation.

### Library preparation and sequencing

Library preparation and sequencing were performed at the WSI Scientific Operations core. Pacific Biosciences HiFi circular consensus DNA sequencing libraries were prepared using the PacBio Express Template Preparation Kit v2.0 (Pacific Biosciences, California, USA) as per the manufacturer's instructions. The kit includes the reagents required for removal of single-strand overhangs, DNA damage repair, end repair/A-tailing, adapter ligation, and nuclease treatment. Library preparation also included a library purification step using AMPure PB beads (Pacific Biosciences, California, USA) and size selection step to remove templates shorter than 3 kb using AMPure PB modified SPRI. DNA concentration was quantified using the Qubit Fluorometer v2.0 and Qubit HS Assay Kit and the final library fragment size analysis was carried out using the Agilent Femto Pulse Automated Pulsed Field CE Instrument and gDNA 165kb gDNA and 55kb BAC analysis kit. Samples were sequenced using the Sequel IIe system (Pacific Biosciences, California, USA). The concentration of the library loaded onto the Sequel IIe was between 40–135 pM. The SMRT link software, a PacBio web-based end-to-end workflow manager, was used to set-up and monitor the run, as well as perform primary and secondary analysis of the data upon completion.

For Hi-C library preparation, DNA was fragmented to a size of 400 to 600 bp using a Covaris E220 sonicator. The DNA was then enriched, barcoded, and amplified using the NEBNext Ultra II DNA Library Prep Kit following manufacturers’ instructions. The Hi-C sequencing was performed using paired-end sequencing with a read length of 150 bp on an Illumina NovaSeq 6000 instrument.

Poly(A) RNA-Seq libraries were constructed using the NEB Ultra II RNA Library Prep kit, following the manufacturer’s instructions. RNA sequencing was performed on the Illumina NovaSeq 6000 instrument.

### Genome assembly, curation and evaluation

***Assembly***

The HiFi reads were assembled using Hifiasm ( Cheng *et al.*, 2021) with the --primary option. Haplotypic duplications were identified and removed using purge_dups ( Guan *et al.*, 2020). The Hi-C reads were mapped to the primary contigs using bwa-mem2 ( Vasimuddin *et al.*, 2019). The contigs were further scaffolded using the provided Hi-C data ( Rao *et al.*, 2014) in YaHS ( Zhou *et al.*, 2023) using the --break option. The scaffolded assemblies were evaluated using Gfastats ( Formenti *et al.*, 2022), BUSCO ( Manni *et al.*, 2021) and MERQURY.FK ( Rhie *et al.*, 2020).

The mitochondrial genome was assembled using MitoHiFi ( Uliano-Silva *et al.*, 2023), which runs MitoFinder ( Allio *et al.*, 2020) and uses these annotations to select the final mitochondrial contig and to ensure the general quality of the sequence.

***Assembly curation***

The assembly was decontaminated using the Assembly Screen for Cobionts and Contaminants (ASCC) pipeline (article in preparation). Manual curation was primarily conducted using PretextView ( Harry, 2022), with additional insights provided by JBrowse2 ( Diesh *et al.*, 2023) and HiGlass ( Kerpedjiev *et al.*, 2018). Scaffolds were visually inspected and corrected as described by Howe *et al*. (2021). Any identified contamination, missed joins, and mis-joins were corrected, and duplicate sequences were tagged and removed. Sex chromosomes were identified by synteny analysis. The entire process is documented at https://gitlab.com/wtsi-grit/rapid-curation (article in preparation).

***Assembly quality assessment***

The Merqury.FK tool ( Rhie *et al.*, 2020), run in a Singularity container ( Kurtzer *et al.*, 2017), was used to evaluate *k*-mer completeness and assembly quality for the primary and alternate haplotypes using the *k*-mer databases ( *k* = 31) that were computed prior to genome assembly. The analysis outputs included assembly QV scores and completeness statistics.

A Hi-C contact map was produced for the final version of the assembly. The Hi-C reads were aligned using bwa-mem2 ( Vasimuddin *et al.*, 2019) and the alignment files were combined using SAMtools ( Danecek *et al.*, 2021). The Hi-C alignments were converted into a contact map using BEDTools ( Quinlan & Hall, 2010) and the Cooler tool suite ( Abdennur & Mirny, 2020). The contact map was visualised in HiGlass ( Kerpedjiev *et al.*, 2018).

The genome was also analysed within the BlobToolKit environment ( Challis *et al.*, 2020) and BUSCO scores ( Manni *et al.*, 2021) were calculated.

Table 4 contains a list of relevant software tool versions and sources.

**Table 4.** Software tools: versions and sources.

Software tool	Version	Source
BlobToolKit	4.2.1	https://github.com/blobtoolkit/blobtoolkit
BUSCO	5.3.2	https://gitlab.com/ezlab/busco
bwa-mem2	2.2.1	https://github.com/bwa-mem2/bwa-mem2
Cooler	0.8.11	https://github.com/open2c/cooler
Gfastats	1.3.6	https://github.com/vgl-hub/gfastats
Hifiasm	0.16.1-r375	https://github.com/chhylp123/hifiasm
HiGlass	1.11.6	https://github.com/higlass/higlass
Merqury	MerquryFK	https://github.com/thegenemyers/MERQURY.FK
MitoHiFi	2	https://github.com/marcelauliano/MitoHiFi
PretextView	0.2	https://github.com/wtsi-hpag/PretextView
purge_dups	1.2.3	https://github.com/dfguan/purge_dups
YaHS	1.2a	https://github.com/c-zhou/yahs

### Genome annotation

The Ensembl Genebuild annotation system ( Aken *et al.*, 2016) was used to generate annotation for the *Argentina silus* assembly (GCA_951799395.1) in Ensembl Rapid Release at the EBI. Annotation was created primarily through alignment of transcriptomic data to the genome, with gap filling via protein-to-genome alignments of a select set of proteins from UniProt ( UniProt Consortium, 2019).

### Wellcome Sanger Institute – Legal and Governance

The materials that have contributed to this genome note have been supplied by a Tree of Life collaborator.

The Wellcome Sanger Institute employs a process whereby due diligence is carried out proportionate to the nature of the materials themselves, and the circumstances under which they have been/are to be collected and provided for use. The purpose of this is to address and mitigate any potential legal and/or ethical implications of receipt and use of the materials as part of the research project, and to ensure that in doing so we align with best practice wherever possible.

The overarching areas of consideration are:

- Ethical review of provenance and sourcing of the material
- Legality of collection, transfer and use (national and international)

Each transfer of samples is undertaken according to a Research Collaboration Agreement or Material Transfer Agreement entered into by the Tree of Life collaborator, Genome Research Limited (operating as the Wellcome Sanger Institute) and in some circumstances other Tree of Life collaborators.

## Data Availability

European Nucleotide Archive: Argentina silus (greater argentine). Accession number PRJEB59966; https://identifiers.org/ena.embl/PRJEB59966. The genome sequence is released openly for reuse. The *Argentina silus* genome sequencing initiative is part of the European Reference Genome Atlas Pilot Project ( https://www.erga-biodiversity.eu/pilot-project). All raw sequence data and the assembly have been deposited in INSDC databases. Raw data and assembly accession identifiers are reported in Table 1 and Table 2.

## Author information

Members of the Wellcome Sanger Institute Tree of Life Management, Samples and Laboratory team are listed here: https://doi.org/10.5281/zenodo.12162482.

Members of Wellcome Sanger Institute Scientific Operations: Sequencing Operations are listed here: https://doi.org/10.5281/zenodo.12165051.

Members of the Wellcome Sanger Institute Tree of Life Core Informatics team are listed here: https://doi.org/10.5281/zenodo.12160324.

Members of the Tree of Life Core Informatics collective are listed here: https://doi.org/10.5281/zenodo.12205391.
