# Supplementary material for: The genome sequence of the greater argentine, Argentina silus (Ascanius, 1775)
Source: Wellcome Open Res. 2025 Mar 17;10:131. [Version 1] doi: 10.12688/wellcomeopenres.23691.1 (PMC12198749; doi:10.12688/wellcomeopenres.23691.1)
Supplement: Peer review history

# Reviewer report: Reviewer response for version 1

**DOI:** 10.21956/wellcomeopenres.26137.r125393

**Published:** 2025-06-25

**Authors:** Jayan Duminda M. Senevirathna

**Affiliations:** 1 Uva Wellassa University, Badulla, Uva Province, Sri Lanka

The article is nearly complete and generally well-written. However, I would like to offer a few suggestions to further strengthen the manuscript.

 Consider including a brief discussion on the importance of genome analysis in the context of conservation genetics. This will help readers understand the broader significance and potential applications of the study.

 Ensure consistent usage of units such as Mb/Kb throughout the manuscript. It would also be helpful to define these abbreviations early in the text, for example: Gb (gigabases), Mb (megabases), Kb (kilobases). 

 Since you have already identified coding genes, it may be valuable to perform a gene set enrichment analysis. This could provide deeper insights into the biological pathways or functional categories represented in the genome, adding further depth to your findings.

Are sufficient details of methods and materials provided to allow replication by others?

Yes

Is the rationale for creating the dataset(s) clearly described?

Yes

Are the datasets clearly presented in a useable and accessible format?

Yes

Are the protocols appropriate and is the work technically sound?

Yes

Reviewer Expertise:

Genetics and Biotechnology

I confirm that I have read this submission and believe that I have an appropriate level of expertise to confirm that it is of an acceptable scientific standard.

# Reviewer report: Reviewer response for version 1

**DOI:** 10.21956/wellcomeopenres.26137.r125398

**Published:** 2025-06-25

**Authors:** Annabel Whibley

**Affiliations:** 1 The University of Auckland, Auckland, Auckland, New Zealand; 2 Grapevine Improvement, Bragato Research Institute, Lincoln, New Zealand

This Data Note reports the genome assembly and annotation of the benthopelagic fish the greater silver smelt ( *Argentina silus). *The introduction sets out the natural history of the species and underscores unresolved questions around the population structure, which would inform fisheries management practice and will be the focus of future population genomics work, aided by this high-quality reference genome.

 HiFi and HiC datasets were generated for assembly purposes. The assembly metrics meet VGP benchmark thresholds. The blobplot does, somewhat unusually, include a cloud of small, low coverage contigs, which I was curious to better understand.  

 Illumina RNAseq data was obtained from gill tissue and used to support Ensembl annotation. I am a little confused by the reporting of the "average transcript length" as >11kb. This is longer than I would expect. Coding sequences, in my understanding, are typically <2kb. If 11kb is truly the average transcript length (and not, for example, the average genomic span) then I would question whether the mean is the correct statistic to be reported here. 

 The Data Note follows Darwin Tree of Life project protocols and reporting templates. Appropriate methods are used, metadata is comprehensive and public accession links are functional. This assembly will provide a valuable foundation for the future study of this species.

Are sufficient details of methods and materials provided to allow replication by others?

Yes

Is the rationale for creating the dataset(s) clearly described?

Yes

Are the datasets clearly presented in a useable and accessible format?

Yes

Are the protocols appropriate and is the work technically sound?

Yes

Reviewer Expertise:

Genomics, Evolution, Bioinformatics

I confirm that I have read this submission and believe that I have an appropriate level of expertise to confirm that it is of an acceptable scientific standard.
